# Dr. Michael Retel

**Published:** 1896-06

**Authors:** 


### Obituary.

Dr. Michael Retel, of Buffalo, died at his residence in this city, May 20, 1896, aged 38 years. He was a native of Buffalo and a son of the late Dr. Caspar Retel. He was a graduate of Buffalo University Medical College, a member of the Medical Society of the County of Erie, and had been engaged in the practice of medicine fifteen years. He is survived by a widow, two brothers and two sisters; one brother, Dr. George Retel, was associated with him in business. Dr. Retel's funeral, held Sunday, May 24th, was largely attended.

